# Effects of different antibacterial disinfectants on microleakage of bulk-fill composite bonded to different tooth structures

**DOI:** 10.1186/s12903-021-01717-7

**Published:** 2021-07-16

**Authors:** Mohammed Bin-Shuwaish, Alhanouf AlHussaini, Lina AlHudaithy, Shamma AlDukhiel, Abdullah AlJamhan, Ali Alrahlah

**Affiliations:** 1 Department of Restorative Dental Sciences, College of Dentistry, King Saud University, P. O. Box 60169, Riyadh, 11545 Kingdom of Saudi Arabia; 2 Department of Pediatric Dentistry, Tufts University School of Dental Medicine, Boston, MA 02111 USA; 3 College of Dentistry, Princess Nourah Bint Abdulrahman University, Riyadh, Kingdom of Saudi Arabia; 4 College of Dentistry, King Saud University, Riyadh, Kingdom of Saudi Arabia; 5 Engineer Abdullah Bugshan Research Chair for Dental and Oral Rehabilitation, College of Dentistry, King Saud University, Riyadh, 11545 Kingdom of Saudi Arabia

**Keywords:** Microleakage, 2% chlorhexidine, Listerine Miswak, Bulk fill, Resin composite, Antibacterial cavity disinfectants, Class V

## Abstract

**Background:**

This in-vitro study aimed to investigate the effect of two different antibacterial disinfectants on the microleakage performance of newly developed bulk-fill composite, bonded to different tooth structures.

**Methods:**

Class V cavities were prepared in 30 sound premolar teeth, with enamel occlusal margins (OM) and dentin cervical margins (CM). Two disinfectants, 2% chlorhexidine gluconate (CHX) and Listerine Miswak (ListM), were used. Teeth were divided into three groups (n = 10): G1, Control; G2, CHX; and G3, ListM. Disinfectants were applied to the cavity preparation walls after they were etched with 35% phosphoric acid. The Single Bond Universal adhesive system was then used, and teeth were restored with Filtek One Bulk Fill composite. Samples were examined, after thermocycling aging, by stereomicroscopy for the evaluation of marginal dye penetration.

**Results:**

The highest mean microleakage score was reported in the CM of G1 (2.60 ± 1.174), which was significant compared with that of G2 only (*p* = 0.02). OM in G1 showed no microleakage, with no significant differences found among groups (χ^2^ = 1.39, *p* = 0.50). No significant differences were reported between G2 and G3 (*p* = 0.45 OM; *p* = 0.17 CM).

**Conclusions:**

Cavity pretreatment with CHX is not significantly different to pretreatment with CHX. In contrast, CHX improved the cervical marginal seal as compare to the control group (G1).

## Background

Composite materials are widely used in restorative dentistry due to their excellent esthetic properties [1, 2]. However, Polymerization shrinkage seems to be the most significant problem with the composite restorations, which may result in microleakage [3, 4], secondary caries, and subsequently may lead to bonding failure [5]. The presence of viable microorganisms that remain after cavity preparation aggravates the problems associated with microleakage, resulting in secondary or residual caries [6]. Incomplete caries removal, and weak adhesion between the restoration and tooth margins are among the main factors responsible for dental restoration failure, allowing the penetration of microorganisms and formation of secondary caries [7]. Studies demonstrated that after the preparation of cavity only a small section of teeth remain disinfected [8]. Therefore, the chances of recurrent dental caries is high which is considered the main cause for replacement of restorations [9]. In Class V restorations, up to 90% of recurrent caries was reported to be at the cervical margins, regardless of the type of restoration [9, 10]. Therefore, improper cleaning of bacterial remnants from the cavity walls during cavity preparation could contaminate the tooth margins and weaken the sealing ability of the restoration, hence leading to unwanted marginal adhesion failure.

The passage of oral fluids, molecules, bacteria, and ions at the interface of the cavity walls and cavity filling material is defined as, microleakage [11–15]. For successful and durable cavity restoration, the deterrence of microleakage is imperative to avoid negative effects such as marginal colouring and fracture, secondary carries, corrosion, sensitivity, and inflammation of the pulp, [12]. These effects are due to the presence of bacteria, their nutrients or hydrogen ions, originating from plaque on the surface, leaking into the interfacial space [16, 17].

The mechanical caries removal techniques are not adequately enough to thoroughly eliminate caries [18]. Therefore, to prevent of secondary caries sequelae, the use of antibacterial disinfectants in conjunction with mechanical approaches is considered an effective method for caries debridement [19]. Chlorhexidine digluconate (CHX) is a powerful antimicrobial solution with high ability to suppress the growth of streptococci, and therefore, has the potential to prevent dental caries [20, 21]. When applied on dentin surface, CHX can prevent bacterial settlement over time due to the continuous release of positively charged molecules [22]. This antimicrobial effect is referred to as substantive antimicrobial activity (SAA) [23]. Barsani et al. found 2% CHX to be a potent disinfectant with effective SAA [23]. Moreover, since the formation of the dentin hybrid layer, during bonding procedure, may not be enough to stabilize the bond over time [24]. The substantive antimicrobial properties of CHX prevents collagen fibrils deterioration after immediate application, and therefore, may maintain a reliable dentin bond [25–27]. Several studies have reported the effects of CHX on the bonding of resin composites to dentin, and these have been found to vary according to different factors, the most important of which are the concentration of CHX, the type of adhesive system, and the timing of CHX application [28–30]. If etch-and rinse systems are used, CHX application has been recommended after acid etching, and before bonding step [31, 32], as the application of the CHX after acid etchant was reported to be highly effective in preserving the adhesive interface [31, 32]. However, studies that evaluated CHX during cavity preparation and its effect on the microleakage were mainly performed on conventional composites, and investigations of their effects and effectiveness on the fairly new bulk fill composites are limited.

Similarly, *Salvadora persica (S. persica)*, Miswak, is a plant species that possesses significant antibacterial properties [33]. The documented natural antibacterial effects of this tree against cariogenic pathogens [34, 35] had made the World Health Organization (WHO) to encourage the use of disinfectants with miswak extracts in the fight against oral bacteria [29]. However, data on the effects of these disinfectants on tooth-colored materials, are scarce.

The aim of this study was to evaluate the impact of various disinfectants (2% CHX and Listerine Miswak antibacterial solutions) on the microleakage performance of a newly developed bulk fill nanocomposite bonded to different tooth structures by using single bond universal adhesive system. The null hypothesis was that no significant difference would be found in the microleakage performance of the bonded tooth structures pretreated with any one of the disinfectants. In addition, there would be no significant difference in the microleakage of pretreated (with disinfectants) and untreated tooth structures.

## Methods

### Teeth collection and sample size calculation

Thirty sound extracted premolar teeth were collected from oral and maxillofacial surgery clinics, and approval was obtained from the ethics committee of the institutional review board (IRB) (Application No. E-18-3312). Teeth with caries lesions, previous restorations, endodontic treatment, or fracture were excluded. Teeth were then cleaned and stored in distilled water at 37 °C until being tested. The storage time for all 30 teeth range from 7 to 10 days.

The sample size was calculated based on the estimated effect size between the study groups according to the literature [36]. In total of ten samples for each group (n = 10) were determined, to detect effect size (f) = 0.656 with type II error (β) = 0.20, at 80% level of power and significant level of 0.05. Sample calculation was conducted using G*Power version 3.1.9.4 (University of Düsseldorf, Germany).

### Tooth preparation

Class V non-beveled cavity preparations (depth, 2 mm; width, 4 mm; height, 3 mm) were prepared on each tooth by means of 330 carbide burs (Columbia, Brasseler, Savannah, GA, USA) mounted in a high-speed handpiece with continues water cooling. Cavities were prepared by one dental clinician and were standardized by drawing the preparation outline on the tooth surface by the aid of customized celluloid matrix centered of the tooth crown. Required dimensions were further verified by a color-coded periodontal probe (Michigan Williams Probe, Hu-Friedy Mfg., Chicago, IL, USA). The occlusal margin was placed in the enamel, while the cervical margin was in the dentin, about 1 mm apical to the cement-enamel junction (CEJ).

Bulk fill nanocomposite (Filtek™ One Bulk Fill, 3M ESPE, St. Paul, MN, USA), Shade A2, was used to restore the preparations. The cavity preparation on each tooth was etched with 35% phosphoric acid (Ultra-Etch®, Ultradent, South Jordan, UT, USA) for 15 s, rinsed for 15 s, and gently air-dried for 5 s, before application of the adhesive bonding system (3M™ Single Bond Universal, 3M ESPE).

Before the teeth were restored with the composite material, two antibacterial disinfectants were used: 2% chlorhexidine gluconate [CHX] (Consepsis® [CHX], Ultradent, South Jordan, UT, USA), and Listerine (LISTERINE® Miswak [ListM], St. Louis, MO, USA). Table 1 shows a list of materials used in the study.

**Table 1:** List of materials used in the study

Material	Company	Composition
FiltekTM One Bulk Fill Posterior Composite Resin Restorative Material Shade A2	3M ESPE, St. Paul, MN, USA	AFM, AUDMA, UDMA, and 1, 12-dodecane-DMA. Fillers: A combination of a 20-nm silica filler, 4- to 11-nm zirconia filler, and a ytterbium trifluoride filler. Inorganic filler: 76.5% by weight (58.5% by volume)
3MTM Single Bond Universal Adhesive Bonding System	3M ESPE, St. Paul, MN, USA	MDP phosphate monomer, HEMA, ethanol, vitrebond copolymer, filler, water, initiators, dimethacrylate resins, and silane
Ultra-Etch® Phosphoric Acid	Ultradent, South Jordan, UT, USA	35% phosphoric acid in water, thickening agent, and colorants
Consepsis® Antibacterial Solution [CHX]	Ultradent, South Jordan, UT, USA	2.0% chlorhexidine gluconate solution
LISTERINE® Miswak Antibacterial Solution [ListM]	Johnson & Johnson Consumer Inc., St. Louis, MO, USA	Aqua, Salvadora persica extract, sorbitol, propylene glycol, poloxamer 407, sodium lauryl sulfate, zinc chloride, benzoic acid, eucalyptol, aroma, sodium benzoate, methyl salicylate, thymol, sodium fluoride, menthol, sodium saccharin, sucralose, glycerin, and sodium fluoride (220 ppm F)

### Study groups

Teeth were randomly divided into three groups, according to the type of surface pretreatment, with 10 teeth in each group (n = 10):

- Group 1 (Control): Teeth were rinsed with distilled water.
- Group 2 (CHX): Teeth were pretreated with 2% Chlorhexidine gluconate.
- Group 3 (ListM): Teeth were pretreated with Listerine Miswak.

Application of disinfectants was performed after teeth were acid-etched and before bonding application, according to the manufacturer’s instructions. Each cavity preparation was restored with a single layer of the bulk fill composite, then light-cured with the light-emitting-diode laser (LED) curing unit of 1200 mW/cm^2^ light intensity (Bluephase® Style, Ivoclar Vivadent, Schaan, Liechtenstein). Restorations were finished with composite diamond finishing burs, then with soflex discs (Sof-Lex®, 3 M ESPE).

### Preparation for microleakage testing

After all restorations were completed, teeth were stored in distilled water at 37 °C for 24 h, after which a thermo-cycling aging procedure was performed for 1500 cycles (5 °C/55 °C) with a dwell time of 30 s and a transfer time of 10 s (Thermocycler THE 1100, SD Mechatronik, Feldkirchen-Westerham, Germany). Tooth surfaces were then covered with two layers of acid-resistant varnish up to about 1 mm from the occlusal and cervical margins of the preparations, and stored in 2% methylene blue solution at room temperature for 24 h. Teeth were then rinsed, gently air-dried, and embedded in a cold-curing orthodontic acrylic resin (Orthoplast, Vertex, Soesterberg, Netherlands) in preparation for being sectioned.

**Fig. 1:**
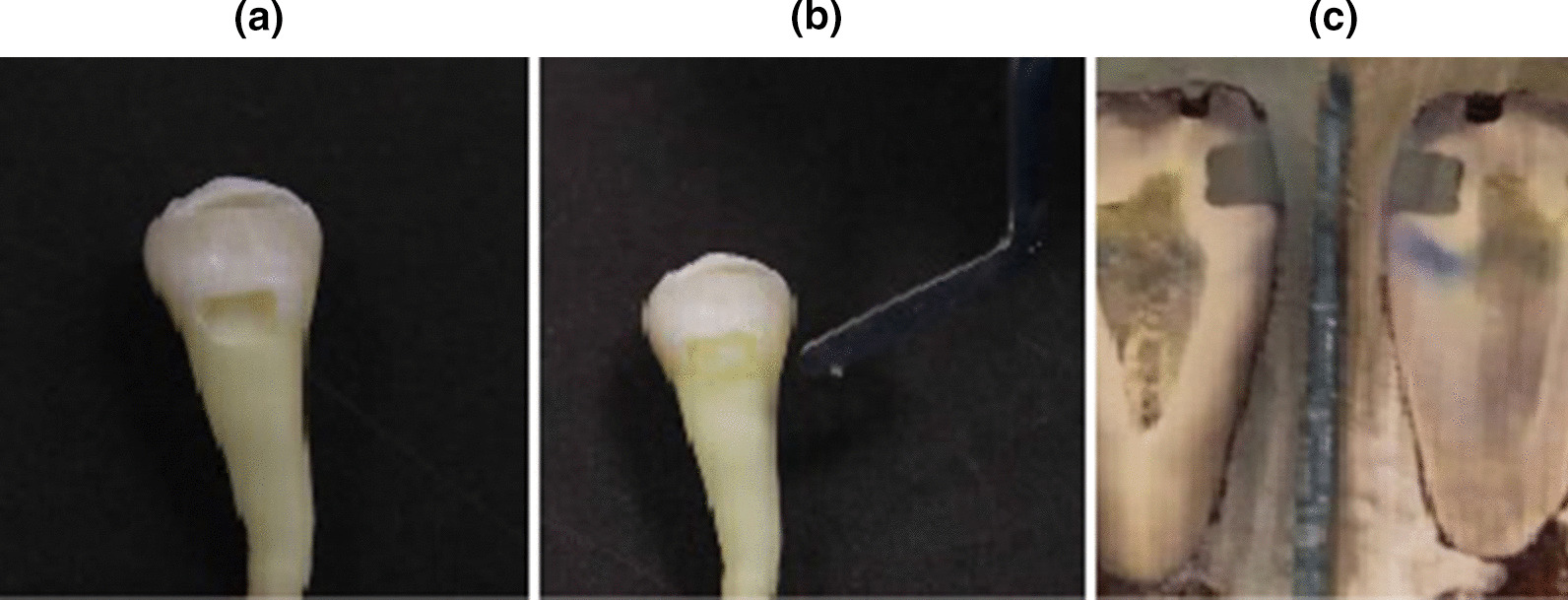
Pictures showing a cavity preparation (**a**), restoration (**b**), and tooth sections before dye penetration scoring

Three longitudinal sections in bucco-lingual direction, one in the center and two lateral sections close to the mesial and distal margins, were created with a precision saw (Isomet 2000, Buehler, Lake Bluff, IL, USA) under water cooling [37, 38] (Fig. 1).

Tooth sections were examined for microleakage on occlusal and cervical margins, by two independent evaluators, using a digital stereomicroscope (HiRoX, Tokyo, Japan) at 50 × magnification, and the most advanced dye penetration among the three sections for each sample was recorded according to the following scoring system:

- 0 = No dye penetration.
- 1 = Penetration less than 1/2 of the cavity wall depth.
- 2 = Penetration up to 1/2 of the cavity wall depth.
- 3 = Penetration more than 1/2 of the cavity wall depth, but not including the pulpal floor.
- 4 = Penetration including the pulpal floor.

### Statistical analysis

Data were collected and statistically analyzed by means of SPSS 12.0 software for Windows (SPSS Inc., Chicago, IL, USA) and a Kruskal–Wallis one-way analysis of variance (ANOVA) test, followed by the Mann–Whitney U test at a significance level of *p* < 0.05.

## Results

**Fig. 2:**
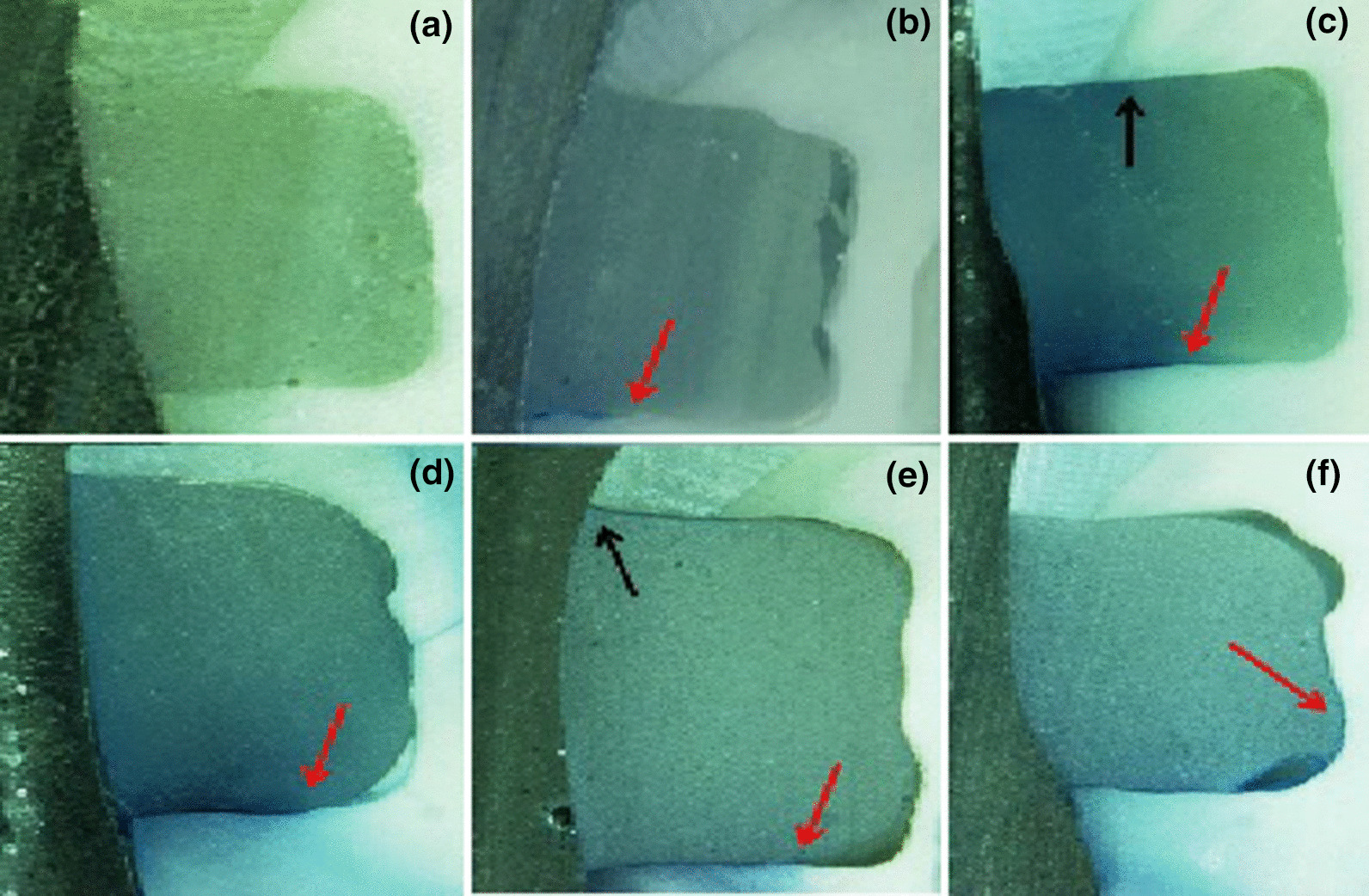
Samples showing different dye penetration scores for occlusal margins (OM) and cervical margins (CM). Black and Red arrows indicate microleakage scores of more than 0 in OM and CM, respectively: **a** CHX-pretreated sample with OM and CM scored 0; **b** CHX-pretreated sample with OM scored 0 and CM scored 1; **c** Listerine M-pretreated sample with OM scored 2 and CM scored 3; **d** control sample with OM scored 0 and CM scored 3; **e** Listerine M-pretreated sample with OM scored 1 and CM scored 3; and **f** control sample with OM scored 0 and CM scored 4

The tooth count recorded for each dye penetration score is presented in Table 2 for each of the three groups studied. Figure 2 shows microscopic images of dye penetration for various group samples.

**Table 2:** Microleakage scores for each tooth margin

Study groups	Occlusal margins score					Cervical margins score				
	0	1	2	3	4	0	1	2	3	4
Control	10	0	0	0	0	0	2	3	2	3
CHX	9	1	0	0	0	4	2	3	1	0
ListM	7	2	1	0	0	0	6	0	3	1

### Group comparisons

#### Control versus disinfectants

**Fig. 3:**
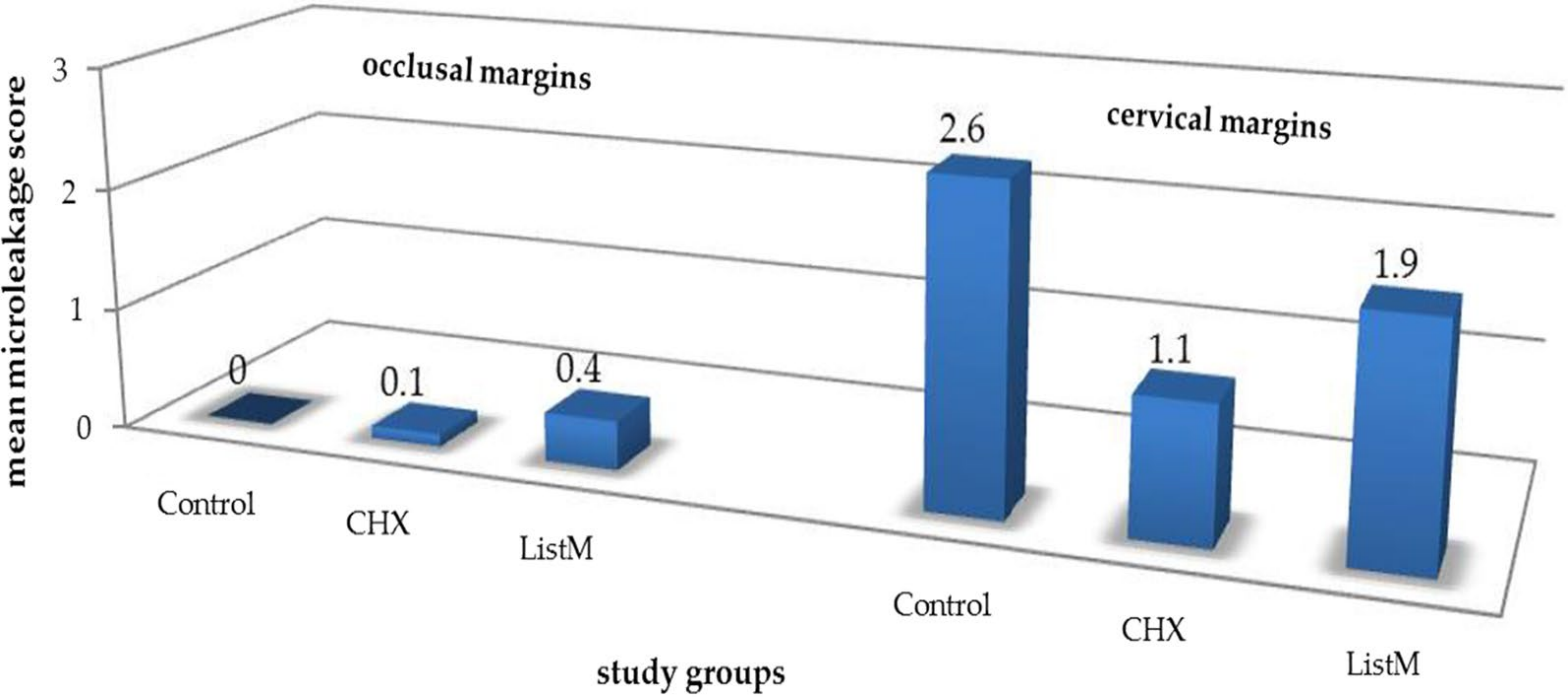
Mean microleakage scores of different tooth structures for the study groups

The highest mean microleakage scores were reported in the cervical margins of the control group (2.60 ± 1.174), followed by ListM (1.90 ± 1.197), and the lowest were recorded in the CHX group (1.10 ± 1.101) for the same margin (Fig. 3).

Kruskal–Wallis one-way ANOVA indicated significant differences among groups (χ^2^ = 6.36, *p* = 0.04) (Table 3). However, the Mann–Whitney test showed significant differences only between control and CHX groups (*p* = 0.02), with no significant differences between control and ListM groups (*p* = 0.2), or between CHX and ListM groups (*p* = 0.17). For the occlusal margins, the control group showed no microleakage (0.00 ± 0.000) compared with groups pretreated with CHX (0.10 ± 0.316) and ListM (0.40 ± 0.699). However, the differences among the three groups were not significant (χ^2^ = 1.39, *p* = 0.5).

**Table 3:** Comparison of disinfectant groups for each tooth structure

Tooth structure	Type of disinfectant Mean microleakage score (± SD)			*df*	Kruskal Wallis *H*	*p* value
	Control	CHX	ListM			
Occlusal	0.00 (± 0.000)	0.10 (± 0.316)	0.40 (± 0.699)	2	1.3942	0.50
Cervical	2.60 (± 1.174)^a^	1.10 (± 1.101)^b^	1.90 (± 1.197)^a,b^	2	6.3252	0.04*

Different lower-case superscript letters indicate statistically significant differences between groups in the same row (*p* < 0.05)

*Statistically significant difference (*p* < 0.05)

#### Chlorhexidine versus Listerine Miswak

Although groups pretreated with ListM showed higher mean microleakage scores in occlusal margins compared with CHX (0.40 ± 0.699 and 0.10 ± 0.316, respectively) and in cervical margins (1.90 ± 1.197 and 1.10 ± 1.101, respectively), the differences were not significant in both tooth structures (*p* = 0.45 for occlusal margins, and *p* = 0.17 for cervical margins).

#### Tooth structure comparisons

In all groups, cervical margins showed significantly higher microleakage than did occlusal margins (*p* = 0.0002, control; *p* = 0.046, CHX; and *p* = 0.005, ListM group) (Table 4).

**Table 4:** Comparison of tooth structure groups for each disinfectant

Study groups	Tooth structures Mean microleakage score (± SD)		Mann–Whitney *U*	*p* value
	Occlusal margins	Cervical margins		
Control	0.00 (± 0.000)	2.60 (± 1.174)	0	0.0002*
CHX	0.10 (± 0.316)	1.10 (± 1.101)	23	0.046*
ListM	0.40 (± 0.699)	1.90 (± 1.197)	12	0.005*

*Statistically significant difference (*p *< 0.05)

## Discussion

Due to the significant antibacterial effects of disinfectants, increasing number of dental clinicians tend to apply them to cavity preparations before proceeding with dental restoration [39]. However, any unwanted interference of these materials with the success of the adhesion procedure of the restorative materials to tooth structures will cause their benefits to diminish. It has been reported in the literature that the techniques used to evaluate the sealing properties of composites by microleakage testing are not different [40]. In the current study, dye-penetration was used due to its well documented results [41]. Further, the microleakage of a recently developed bulk-fill composite bonded to Class V cavity preparations after pretreatment with 2% CHX and Listerine Miswak disinfectants was evaluated. A non-beveled preparation was used in an effort to standardize the preparations in the examined teeth, since it has been documented that beveling of enamel has no beneficial effect on either retention or marginal discoloration [42, 43].

The results of this study, revealed the superior sealing ability of the occlusal margins, against microleakage, compared with that in cervical margins, regardless of the use of disinfectant, when etch-and-rinse mode of the universal bond system was used. These results were in agreement with those of Yamauchi and co-workers, since they found that a separate etching step prior to the application of universal adhesive system resulted in better enamel marginal integrity [44]. This can be explained by the high percentage content of hydroxyapatite inorganic contents in enamel compared to dentin which contains higher amount of organic structures, and therefore, may interfere with the micromechanical adhesion procedure [45]. Several studies reported similar results on the effects of etch-and-rinse systems on both tooth structures [43, 46, 47]. In the current study, significantly increased microleakage, in cervical margins, compared to occlusal margins was found before and after pretreatment with both types of disinfectants. Therefore, the null hypothesis, that there would be no significant difference in microleakage between different tooth structures pretreated with different disinfectants, was rejected for all groups. The results revealed that, pretreatment of tooth structures with CHX or Listerine Miswak did not adversely affect the marginal seal of the restoration. These results were in agreement with those of multiple studies that evaluated CHX as a pretreatment disinfectant and found no significant effect on microleakage, regardless of the type of bonding system used [48–50]. In both occlusal and cervical margins, CHX-pretreated teeth showed better marginal seal than those treated with Listerine Miswak; however, this difference was not statistically significant in both tooth structures. Therefore, the first null hypothesis, that there would be no significant difference in microleakage between disinfectants, cannot be rejected for both margin types. It is worth mentioning here that a statistically non-significant difference between the two disinfectants may not reflect their real effects in the case of a small sample size. In other words, increasing the power of the analysis by increasing the sample size may render this significant difference. The improvement in the cervical margins of CHX-pretreated specimens may be explained by the inherited properties of CHX to act as a matrix metalloproteinase inhibitor, which may prevent collagen destruction at the bonding interface [51]. Therefore, CHX application has been shown to maintain the dentin hybrid layer preservation [25, 26, 52]. In contrast, miswak extracts have been documented to disrupt the smear layer on dentin [34]. Recently, Khunkar et al. [53] compared the effects of miswak natural extracts with those of CHX on dentin collagen. The results revealed that, miswak extracts were found to have the ability to prevent collagen degradation at a lower effect than CHX. To the best of the authors knowledge, only one study (conducted by Salama et al.,) [36] has been found, where the effects of miswak extracts on microleakage were evaluated. The study reported no significant difference in microleakage of Class V conventional resin composites bonded to etched dentin margins of primary teeth pretreated with 1 mg/mL extracts of miswak or CHX. However, the CHX concentration they used was 0.2%. Listerine Miswak, used in the current study, is a chemical disinfectant with different components, other than miswak extracts. Therefore, its effect on microleakage, is attributed to the effect of the disinfectant rather than the miswak extract by itself. CHX has been recommended for use on etched dentin, with etch-and-rinse adhesives [54].

Universal adhesive system was used in the “etch-and-rinse” mode, which may explain the superior marginal sealing results in occlusal structure of the tooth, where all margins are in enamel. Conversely, cervical margins presented significantly high microleakage scores in all groups, especially in non-pretreated specimens. This could be attributed to the increased etching depth on the dentin margins that cannot be completely infiltrated with resin and yielded a weak bond which subsequently resulted in increased microleakage. However, when these margins were pretreated with either type of disinfectant, sealing abilities improved, which was significant in the CHX group only. Therefore, the second null hypothesis, that there would be no significant difference in microleakage between pretreated and non-pretreated groups, was rejected for the cervical margins of the CHX group.

Multiple studies reported the effectiveness of CHX in stabilizing the bond strength and improves the adhesion of composite restorations to dentin over time [26, 55, 56]. Carrilho et al. [26] reported that dentin pretreatment with CHX by etch-and-rinse systems was found to stabilize the bond after 14 months. Similarly, Ekambaram et al. reported that CHX maintained the bond strength of composite to tooth structures and decreased leakage after 1 year [55]. Komori et al. [50], studied the application of 2% CHX with etch-and-rinse adhesive systems and revealed that, CHX preserve the bond strength to sound dentin for up to 6 months. However, some studies reported no effect of CHX on long-term bond preservation [57, 58]. A study conducted by Angeloni et al. [58], showed no effect of CHX on the dentin bond with a self-adhesive system after 6 to 12 months. Similarly, Simões et al. [57] revealed that CHX application with an etch-and-rinse adhesive system had no effect on the sealing performance after 6 months. In a recently published meta-analysis, the sum report for the dentin bond strength after 6 months evaluations was found to be improved [59]. The results from the current study revealed that, the application of CHX to enamel did not improve the bond strength to composite restorations. These results were in agreement with previous studies by Kapdan et al. and Haidari et al.[60, 61], who used “etch-and-rinse” systems. Haidari et al., [61] used the same adhesive agent protocol used in the current study and found no effect of CHX on enamel microleakage. This may be explained by the high stability of bond to inorganic enamel before the application of disinfectant, as compared with that of dentin. It is worth mentioning that, majority of the studies evaluated the effects of CHX on bonding conventional composites to different tooth structures. Bulk-fill composites were not fully evaluated in that regard.

Although marginal integrity is not the only factor to determine the influence of certain clinical procedure or protocol during restorative treatment, microleakage is considered a common laboratory test to predict the longevity of dental restorations. However, in comparison with bacterial penetration, the characteristics of the molecular dyes used to detect microleakage may allow them to penetrate differently in spaces. Therefore, clinical correlations are necessary to validate these methods of assessment [62]. Clinical researches is believed to be the most appropriate method to evaluate the longevity of dental materials. However, clinical researches are difficult to standardize. In addition, they require continuous and long follow up to evaluate the performance of dental restorations during function [40].

In-vitro studies tend to test hypotheses in a controlled laboratory set-up. One of the measures applied in laboratory studies, to simulate clinical situations, is the artificial aging process which simulates the oral cavity environment.

### Limitations of the study

The current study evaluated the short-term effects of disinfectant pretreatment on bonding to Class V restorations subjected to an artificial aging protocol with thermal challenges. The main limitation of this study is the lack of statistical power due to a relatively small sample size. Kruskal–Wallis one-way ANOVA indicated significant differences among three groups, however posc hoc analysis only revealed statistical difference between control and CHX group. The limitation of small sample size implies that further research is needed to confirm these findings. Wahab et al. [63] reported that thermocycling between 5 and 55 °C for 500 cycles significantly increased microleakage in Class V restorations; however, other studies did not report significant effects of thermocycling on microleakage [64, 65]. Furthermore, only one composite material was evaluated in this study, using different types of restorative materials, to bond to tooth structures, and compare results with bulk fill composites would add to the values of the bond evaluation. For the newly developed adhesion systems, to bond bulk-fill composite to different tooth structures, particularly enamel, only trace information is available on the effect of CHX, and none for the miswak extracts, on the long-term bond strength. Therefore, more controlled studies to evaluate their long-term effects are warranted.

### Clinical implications

In the current study, the absence of negative effects on dentin leakage may indicate that the protocol of cleansing the cavity margins, during cavity preparation, with CHX or Listerine Miswak disinfectants was not harmful to the bond integrity after short time of simulated function.

## Conclusions

Within the limitations of this study, it could be concluded that, Pretreatment of cavity preparations with 2% CHX or Listerine Miswak did not affect the microleakage between Filtek One bulk-fill composite material and different tooth structures bonded with the “etch-and-rinse” mode of the single bond universal adhesive system. On the contrary, pretreatment of the cervical margins with 2% CHX significantly improved the marginal seal.

## Data Availability

The datasets generated and/or analyzed during the current study are not publicly available due [concerns of the participants] but are available from the corresponding author on reasonable request.

## Abbreviations

OM: Occlusal margins
CM: Cervical margins
CHX: Chlorhexidine gluconate
ListM: Listerine Miswak
SAA: Substantive antimicrobial activity
CEJ: Cement-enamel junction
LED: Light-emitting-diode laser
ANOVA: Analysis of variance

## References

[1] Jun S-K, Kim D-A, Goo H-J, Lee H-H (2013). Investigation of the correlation between the different mechanical properties of resin composites. Dent Mater J.

[2] AlJehani YA, Baskaradoss JK, Geevarghese A, AlShehry MA, Vallittu PK (2015). Shear bond strength between alumina substrate and prosthodontic resin composites with various adhesive resin systems. BMC Oral Health.

[3] Ferracane JL (2011). Resin composite–state of the art. Dent Mater.

[4] Cheung GS (1990). Reducing marginal leakage of posterior composite resin restorations: a review of clinical techniques. J Prosthet Dent.

[5] Singla M, Aggarwal V, Kumar N (2011). Effect of chlorhexidine cavity disinfection on microleakage in cavities restored with composite using a self-etching single bottle adhesive. J Conserv Dent JCD.

[6] Say EC, Koray F, Tarim B, Soyman M, Gülmez T (2004). In vitro effect of cavity disinfectants on the bond strength of dentin bonding systems. Quintessence Int.

[7] Gagliardi RM, Avelar RP (2002). Evaluation of microleakage using different bonding agents. Oper Dent.

[8] Brännström M (1986). The cause of postrestorative sensitivity and its prevention. J Endod.

[9] Ferracane JL (2017). Models of caries formation around dental composite restorations. J Dent Res.

[10] Mjor IA (1998). The location of clinically diagnosed secondary caries. Quintessence Int (Berlin, Germany: 1985).

[11] Güneş Ş, Bahsi E, İnce B, Çolak H, Dalli M, Yavuz İ, Sahbaz C, Cangül S (2014). Comparative evaluation of the effects of ozone, diode laser, and traditional cavity disinfectants on microleakage. Ozone Sci Eng.

[12] Larson TD. The clinical significance and management of microleakage. Part one. Northw Dent. 2005; 84(1):23–25, 28. 15807140

[13] Vicente A, Ortiz AJ, Bravo LA (2009). Microleakage beneath brackets bonded with flowable materials: effect of thermocycling. Eur J Orthod.

[14] Gogna R, Jagadis S, Shashikal K (2011). A comparative in vitro study of microleakage by a radioactive isotope and compressive strength of three nanofilled composite resin restorations. J Conserv Dent JCD.

[15] Zhang Y, Chen W, Zhang J, Li Y (2020). Does Er, Cr:YSGG reduce the microleakage of restorations when used for cavity preparation? A systematic review and meta-analysis. BMC Oral Health.

[16] Ferrari M, Garcia-Godoy F (2002). Sealing ability of new generation adhesive-restorative materials placed on vital teeth. Am J Dent.

[17] Pashley DH (1990). Clinical considerations of microleakage. J Endod.

[18] Cheng L, Zhang K, Weir MD, Liu H, Zhou X, Xu HH (2013). Effects of antibacterial primers with quaternary ammonium and nano-silver on *Streptococcus mutans* impregnated in human dentin blocks. Dent Mater.

[19] Brännström M (1987). Infection beneath composite resin restorations: can it be avoided?. Oper Dent.

[20] Bin-Shuwaish MS (2016). Effects and effectiveness of cavity disinfectants in operative dentistry: a literature review. J Contemp Dent Pract.

[21] Rudolf J-L, Moser C, Sculean A, Eick S (2019). In-vitro antibiofilm activity of chlorhexidine digluconate on polylactide-based and collagen-based membranes. BMC Oral Health.

[22] Emilson CG (1977). Susceptibility of various microorganisms to chlorhexidine. Scand J Dent Res.

[23] Basrani B, Santos JM, Tjäderhane L, Grad H, Gorduysus O, Huang J, Lawrence HP, Friedman S (2002). Substantive antimicrobial activity in chlorhexidine-treated human root dentin. Oral Surg Oral Med Oral Pathol Oral Radiol Endod.

[24] Perdigão J, Reis A, Loguercio AD (2013). Dentin adhesion and MMPs: a comprehensive review. J Esthet Restor Dent.

[25] Brackett WW, Tay FR, Brackett MG, Dib A, Sword RJ, Pashley DH (2007). The effect of chlorhexidine on dentin hybrid layers in vivo. Oper Dent.

[26] Carrilho MR, Geraldeli S, Tay F, de Goes MF, Carvalho RM, Tjäderhane L, Reis AF, Hebling J, Mazzoni A, Breschi L (2007). In vivo preservation of the hybrid layer by chlorhexidine. J Dent Res.

[27] Breschi L, Cammelli F, Visintini E, Mazzoni A, Vita F, Carrilho M, Cadenaro M, Foulger S, Mazzoti G, Tay FR (2009). Influence of chlorhexidine concentration on the durability of etch-and-rinse dentin bonds: a 12-month in vitro study. J Adhes Dent.

[28] Di Hipólito V, Rodrigues FP, Piveta FB, Azevedo Lda C, Bruschi Alonso RC, Silikas N, Carvalho RM, De Goes MF, Perlatti D'Alpino PH (2012). Effectiveness of self-adhesive luting cements in bonding to chlorhexidine-treated dentin. Dent Mater.

[29] Halawany HS (2012). A review on miswak (*Salvadora persica*) and its effect on various aspects of oral health. Saudi Dent J.

[30] Zheng P, Zaruba M, Attin T, Wiegand A (2015). Effect of different matrix metalloproteinase inhibitors on microtensile bond strength of an etch-and-rinse and a self-etching adhesive to dentin. Oper Dent.

[31] Komori PC, Pashley DH, Tjäderhane L, Breschi L, Mazzoni A, de Goes MF, Wang L, Carrilho MR (2009). Effect of 2% chlorhexidine digluconate on the bond strength to normal versus caries-affected dentin. Oper Dent.

[32] Ricci HA, Sanabe ME, Costa CA, Hebling J (2010). Effect of chlorhexidine on bond strength of two-step etch-and-rinse adhesive systems to dentin of primary and permanent teeth. Am J Dent.

[33] Al-Sohaibani S, Murugan K (2012). Anti-biofilm activity of *Salvadora persica* on cariogenic isolates of *Streptococcus mutans*: in vitro and molecular docking studies. Biofouling.

[34] Sofrata AH, Claesson RL, Lingström PK, Gustafsson AK (2008). Strong antibacterial effect of miswak against oral microorganisms associated with periodontitis and caries. J Periodontol.

[35] El-Latif Hesham A, Alrumman SA (2016). Antibacterial activity of Miswak Salvadora persica extracts against isolated and genetically identified oral cavity pathogens. Technol Health Care.

[36] Salama F, Balto H, Al-Yahya F, Al-Mofareh S (2015). The effect of cavity disinfectants on microleakage of composite restorations in primary teeth. Eur J Paediatr Dent.

[37] Ferrari M, García-Godoy F (2002). Sealing ability of new generation adhesive-restorative materials placed on vital teeth. Am J Dent.

[38] Deliperi S, Bardwell DN, Wegley C (2007). Restoration interface microleakage using one total-etch and three self-etch adhesives. Oper Dent.

[39] Gunaydin Z, Yazici AR, Cehreli ZC (2016). In vivo and in vitro effects of chlorhexidine pretreatment on immediate and aged dentin bond strengths. Oper Dent.

[40] Moazzami SM, Sarabi N, Hajizadeh H, Majidinia S, Li Y, Meharry MR, Shahrokh H (2014). Efficacy of four lining materials in sandwich technique to reduce microleakage in class II composite resin restorations. Oper Dent.

[41] Raskin A, D'Hoore W, Gonthier S, Degrange M, Déjou J (2001). Reliability of in vitro microleakage tests: a literature review. J Adhes Dent.

[42] Mahn E, Rousson V, Heintze S (2015). Meta-analysis of the influence of bonding parameters on the clinical outcome of tooth-colored cervical restorations. J Adhes Dent.

[43] Loguercio AD, Luque-Martinez IV, Fuentes S, Reis A, Muñoz MA (2018). Effect of dentin roughness on the adhesive performance in non-carious cervical lesions: a double-blind randomized clinical trial. J Dent.

[44] Yamauchi K, Tsujimoto A, Jurado CA, Shimatani Y, Nagura Y, Takamizawa T, Barkmeier WW, Latta MA, Miyazaki M (2019). Etch-and-rinse vs self-etch mode for dentin bonding effectiveness of universal adhesives. J Oral Sci.

[45] Li Q, Jepsen S, Albers HK, Eberhard J (2006). Flowable materials as an intermediate layer could improve the marginal and internal adaptation of composite restorations in Class-V-cavities. Dent Mater.

[46] Pamir T, Türkün M (2005). Factors affecting microleakage of a packable resin composite: an in vitro study. Oper Dent.

[47] Fukegawa D, Hayakawa S, Yoshida Y, Suzuki K, Osaka A, Van Meerbeek B (2006). Chemical interaction of phosphoric acid ester with hydroxyapatite. J Dent Res.

[48] Mobarak EH, El-Korashy DI, Pashley DH (2010). Effect of chlorhexidine concentrations on micro-shear bond strength of self-etch adhesive to normal and caries-affected dentin. Am J Dent.

[49] Mobarak EH (2011). Effect of chlorhexidine pretreatment on bond strength durability of caries-affected dentin over 2-year aging in artificial saliva and under simulated intrapulpal pressure. Oper Dent.

[50] Loguercio AD, Stanislawczuk R, Malaquias P, Gutierrez MF, Bauer J, Reis A (2016). Effect of minocycline on the durability of dentin bonding produced with etch-and-rinse adhesives. Oper Dent.

[51] Pashley DH, Tay FR, Yiu C, Hashimoto M, Breschi L, Carvalho RM, Ito S (2004). Collagen degradation by host-derived enzymes during aging. J Dent Res.

[52] Breschi L, Mazzoni A, Nato F, Carrilho M, Visintini E, Tjäderhane L, Ruggeri A, Tay FR, Dorigo Ede S, Pashley DH (2010). Chlorhexidine stabilizes the adhesive interface: a 2-year in vitro study. Dent Mater.

[53] Khunkar S, Hariri I, Alsayed E, Linjawi A, Khunkar S, Islam S, Bakhsh TA, Nakashima S. Inhibitory effect of Salvadora persica extract (Miswak) on collagen degradation in demineralized dentin: in vitro study. J Dent Sci. 2020. 10.1016/j.jds.2020.05.025 PMC7770310 33384799

[54] Breschi L (2013). Chlorhexidine application to stabilize the adhesive interface: why and how?. J Adhes Dent.

[55] Ekambaram M, Yiu CK, Matinlinna JP, King NM, Tay FR (2014). Adjunctive application of chlorhexidine and ethanol-wet bonding on durability of bonds to sound and caries-affected dentine. J Dent.

[56] Tjäderhane L (2015). Dentin bonding: can we make it last?. Oper Dent.

[57] Simões DM, Basting RT, Amaral FL, Turssi CP, França FM (2014). Influence of chlorhexidine and/or ethanol treatment on bond strength of an etch-and-rinse adhesive to dentin: an in vitro and in situ study. Oper Dent.

[58] Angeloni V, Mazzoni A, Marchesi G, Cadenaro M, Comba A, Maravi T, Scotti N, Pashley DH, Tay FR, Breschi L (2017). Role of chlorhexidine on long-term bond strength of self-adhesive composite cements to intraradicular dentin. J Adhes Dent.

[59] Hamdan-Nassar T, Bellot-Arcís C, Paredes-Gallardo V, García-Sanz V, Pascual-Moscardó A, Almerich-Silla JM, Montiel-Company JM (2019). Effect of 2% chlorhexidine following acid etching on microtensile bond strength of resin restorations: a meta-analysis. Medicina (Kaunas).

[60] Kapdan A, Öztaş N (2015). Effects of chlorhexidine and gaseous ozone on microleakage and on the bond strength of dentin bonding agents with compomer restoration on primary teeth. J Dent Sci.

[61] Haidari M, Abolghasemzade F, Alaghemand H, Esmaeili B (2017). Effect of 2% chlorhexidine on the enamel microleakage of composite restorations using 5th, 6th, 7th and universal generation of dentine bonding agents (in vitro). J Babol Univ Med Sci.

[62] Goldstein RE, Lamba S, Lawson NC, Beck P, Oster RA, Burgess JO (2017). Microleakage around class V composite restorations after ultrasonic scaling and sonic toothbrushing around their margin. J Esthet Restor Dent.

[63] Wahab FK, Shaini FJ, Morgano SM (2003). The effect of thermocycling on microleakage of several commercially available composite Class V restorations in vitro. J Prosthet Dent.

[64] Wendt SL, McInnes PM, Dickinson GL (1992). The effect of thermocycling in microleakage analysis. Dent Mater.

[65] Prati C, Tao L, Simpson M, Pashley DH (1994). Permeability and microleakage of Class II resin composite restorations. J Dent.

